# Hyper-Acute Stroke Systems of Care and Workflow

**DOI:** 10.1007/s11910-024-01367-6

**Published:** 2024-08-16

**Authors:** Timothy J. Kleinig, Patrick McMullan, Geoffrey C. Cloud, Prof Christopher Bladin, Anna Ranta

**Affiliations:** 1 Department of Neurology, Royal Adelaide Hospital, 1 Port Road, Adelaide, South Australia 5000 Australia; 2 Department of Neurology, Alfred Health, Melbourne, VIC Australia; 3 Department of Neuroscience, School of Translational Medicine, Monash University, Melbourne, VIC Australia; 4 Ambulance Victoria/Monash University, Melbourne, Australia; 5 Department of Neurology, Wellington Hospital, Wellington, New Zealand; 6 Department of Medicine, University of Otago, Wellington, New Zealand

**Keywords:** Stroke, Telemedicine, Systems of care, Learning health care system, Geographical disparity

## Abstract

**Purpose of review:**

Recent stroke treatment advances have necessitated agile, broad-scale healthcare system redesign, to achieve optimal patient outcomes and access equity. Optimised hyperacute stroke care requires integrated pre-hospital, emergency department, stroke specialist, radiology, neurosurgical and endovascular neurointervention services, guided by a population-wide needs analysis. In this review, we survey system integration efforts, providing case studies, and identify common elements of successful initiatives.

**Recent findings:**

Different regions and nations have evolved varied acute stroke systems depending on geography, population density and workforce. However, common facilitators to these solutions have included stroke unit care as a foundation, government-clinician synergy, pre-hospital pathway coordination, service centralisation, and stroke data guiding system improvement. Further technological advantages will minimize the geographical distance disadvantages and facilitate virtual expertise redistribution to remote areas.

**Summary:**

Continued treatment advances necessitate an integrated, adaptable, population-wide trans-disciplinary approach. A well-designed clinician-led and government-supported system can facilitate hyperacute care and scaffold future system enhancements.

**Supplementary Information:**

The online version contains supplementary material available at 10.1007/s11910-024-01367-6.

## Introduction

The ethical concepts of justice and beneficence, embodied as ‘What we owe to each other’[1], require that those responsible for stroke systems of care (both governments and clinicians) work collaboratively and flexibly to create and refine systems which can achieve both equity and excellence.

**Fig. 1:**
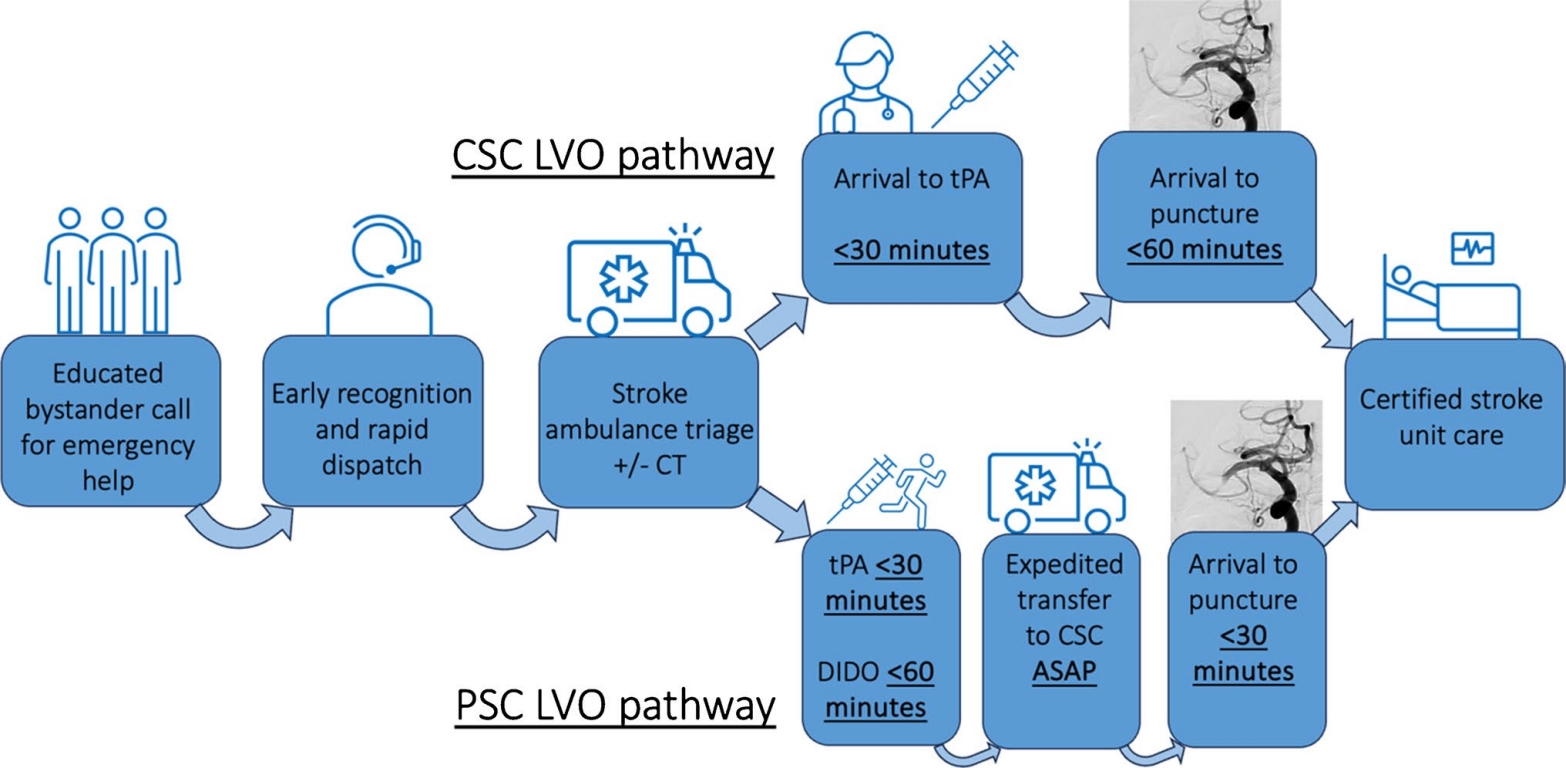
Idealised LVO Stroke pathway for Comprehensive and Primary Stroke Centre. Common idealised elements regardless of patient initial hospital presentation include an immediate call to emergencies services by an educated ‘F.A.S.T.’ (Face, Arm, Speech, Time)-aware bystander, followed by rapid ambulance despatch facilitated by the call-taker. Ambulance services extract the patient quickly, and (if CT-enabled) can perform on-site neuroimaging, distinguish ischaemic stroke from ICH, identify large vessel occlusion, and administer thrombolytic and/or other acute stroke treatments as indicated, assisted potentially by telemedicine. If taken to the CSC, either through proximity or due to suspected or proven LVO, then the patient should be taken straight to the CT on the ambulance stretcher, with thrombolytic administered within 30 min of arrival. The EVT team should be notified immediately once LVO is demonstrated (or highly suspected) and mobilise rapidly to perform thrombectomy within an hour of arrival. If taken to the PSC, thrombolytic should again be administered within 30 min of arrival, with CSC contacted promptly if LVO is detected. As the ambulance crew has taken the patient directly to the CT scanner, the same ambulance bed and crew (if transporting via road) should then promptly take the patient to the CSC, departing within 60 min of arrival, and transporting the patient directly to the EVT suite, where EVT can be performed immediately, as the interventionalist has been mobilised while the patient is en route. All patients should then receive certified stroke unit care. CT = Computed Tomography, tPA = tissue Plasminogen Activator. CSC = Comprehensive Stroke Centre, PSC= Primary Stroke Centre, DIDO = Door-In-Door-Out.

As a health priority, the optimisation of these systems of care is vital, due to both the disease burden [2] and the individual, societal and health economic benefits of ideal care. The time-dependent pathophysiology of both ischaemic stroke and intracerebral haemorrhage requires timely access to stroke expertise. An optimized stroke system of care enables this by addressing community symptom recognition and response, prompt emergency services response, and coordinated pre-hospital and hospital treatment, facilitated by prompt neuroimaging – either in-ambulance or in-hospital (Fig. 1). This has been neatly conceptualised as the stroke ‘chain of survival’, analogous to cardiac arrest resuscitation system improvement approaches [3]. However, the complexity of stroke care systems, and the recent rapid evolution of evidence – especially in the last decade—has made system improvement challenging.

### Historical overview of stroke milestones requiring system design

Stroke units have been the cornerstone and focus of improvements in stroke care, providing concentrated expertise which has facilitated testing of novel treatments through randomised trials. Although the first description of organised stroke systems of care were published in the 1950s, it was a full four decades until the benefits of stroke unit care were convincingly proven through systematic review and meta-analysis of randomised controlled trials [4]. Another decade later stroke unit care was widely affirmed as a healthcare right [5, 6]. However, still too few people with stroke can access stroke unit care even in many developed countries, although in some (e.g. Sweden and the UK) rates exceed 90% [7].

In 1995 the pivotal National Institute for Neurological Disorders trial proved the benefits of alteplase thrombolysis [8], but uptake was slow, in part due to resistance to change, but also due to the complex system improvements required. Nevertheless, these redesigned systems – comprising both hospital-based thrombolytic services and remote expert telestroke services [9]—provided a platform for the investigation of Endovascular stroke Therapies (EVT) and implementation of EVT once proven [10].

Implementation of EVT services occurred rapidly in especially some urbanised high income countries, but less rapidly in other location [11]. However, even regions with rapidly adapting systems have been continually challenged by further scientific advances, including the expansion of the EVT window to 24 h [12] (and possibly beyond) [13], initially in patients with a large volume of salvageable ischaemic brain tissue, but more recently also for patients with basilar occlusion [14, 15] and large core hemispheric infarction [16]. Further, the thrombolytic treatment window has expanded to nine hours [17] and now possibly 24 h [18]. And while the maxim of ‘time is brain’ still holds—the sooner reperfusion therapy can be delivered the better—these extended time frames of potential eligibility have increased the complexity of assessment and volumes of patients requiring hyper-urgent expert assessment. In addition, intracerebral haemorrhage has been shown to require urgent expert treatment, including blood pressure-lowering and reversal of coagulopathy [19]. Minimally invasive surgery for lobar intracerebral haemorrhage for patients within 24 h of stroke onset is now also probably beneficial in centres with appropriate expertise [20].

As noted in an earlier review article in this year’s collection, in large urban centres with appropriate resources, mobile stroke units (CT-capable ambulance which can deliver thrombolytic treatment) are also increasingly integrated into stroke systems, given the benefit in treating not only ischaemic stroke with expedited thrombolysis [21, 22], but probably also intracerebral haemorrhage with intensive blood pressure lowering [23].

As a result of these advances, the majority of patients with moderate to severe acute stroke symptoms are at least potentially eligible for thrombolytic, endovascular and possibly neurosurgical therapies, even if situated many hundreds of kilometres distant from specialist stroke centres [24]. In the next section, we present several examples of stroke care system responses to emerging hyperacute evidence.

## Stroke care system design case studies

### The London Hyperacute Stroke Unit (HASU) – a centralised metropolitan model

The United Kingdom National Health Service provides universal health care to all citizens, through an integrated system of primary care and hospital-based care.

In 2010, acute stroke services were centralised in London from 30 hospitals to 8, in a city at that time of 8.17 million people distributed over 1,572km^2^ [25]. These hospitals were selected both on capability to deliver evidence-based acute stroke care, and the geographical incidence of stroke in the greater London area, with the aim that no patient was to have a transfer time of more than 30 min. Patients were assessed by expert teams 24/7, with immediate access to neuroimaging and reperfusion (at that stage only thrombolytic treatment). Stroke Key Performance Indicators (KPI) were measured by the Sentinel Improvement National Audit Programme (SINAP) (now Sentinel Stroke National Audit Programme (SSNAP)). Centralisation was associated with a significant reductions in mortality (an absolute reduction of 1.1% at 90 days) and length of stay, which was sustained over time [26]. System redesign was highly cost-effective.

This stood in contrast with the contemporaneous system redesign which occurred in Manchester (population 2.68 M distributed over 1,277km^2^). Here, centralisation to one of three centres (one Comprehensive Stroke Centre and 2 Primary Stroke Centres) occurred only for patients with suspected stroke < 4 h since symptoms onset. All other patients were seen in one of ten acute general hospitals. Fidelity to intended design was suboptimal; only 64% of patients with stroke < 4 h since onset were transferred to these HASUs (compared with 98% fidelity in London). Adherence to other quality care measures was also lower. Although length of stay decreased, this system was not associated with a reduction in mortality [25]. Subsequent adoption of the London model (centralisation of all acute stroke presentations) was associated with a reduction in mortality, as well as further reductions in length of stay [26]. During this intervention the proportion of patients admitted to a HASU increased from 39 to 86% and other care metrics trended towards improvement.

More recently, telemedicine in transit provided to stroke patients to assist in triage and hasten reperfusion treatments [27] has been implemented [28].

### The Victorian Stroke Telemedicine (VST) Service – a statewide telestroke service

The Australian health system provides universal health care under a federated model. The Federal Government funds primary care and ambulatory specialist care, and State Governments run public hospitals and ambulance services, co-funded by the federal government. In addition, there is a strong private health sector, partly funded by a government private health rebate, although private hospitals do not commonly establish stroke units due to insufficient financial incentive.

The population of Victoria comprises roughly 6.5 million people, 5 million of whom live in close proximity to an urban Primary or Comprehensive Stroke Centre in the Greater Melbourne area (around 10,000km^2^). The remaining 1.5 million live in regional Victoria and are served by the Victorian Stroke Telemedicine (VST) service (Figure S1).

The VST first commenced in 2009 but was fully established in 2013. It was progressively expanded to its current Victorian extent in 2018 [29]. The 22 VST hospitals (19 in Victoria, 3 in Tasmania) were strategically chosen on the basis of size and geography, such that no patient need travel more than an hour to access a Telestroke-capable hospital. All centres support 24/7 multimodal CT imaging.

Following ‘code stroke’ assessment and imaging, calls are placed via a single number to a call centre, where details are taken and the call assigned to the VST Neurologist. The Neurologist accesses brain imaging which is transferred from the 19 sites to a single imaging platform, and a telestroke consult is established using VIMED TELEDOC 5 HD + telemedicine carts. Consults are recorded on a purpose-built platform which automatically records necessary stroke KPI data for the national stroke registry AuSCR (Australian Stroke Clinical Registry). Patients requiring thrombectomy or neurosurgical intervention are transferred to a metropolitan Comprehensive Stroke Centre.

All VST consultants are Vascular Neurology trained. The day is split into 4 shifts, 0800–1300, 1300–1800, 1800–2400 and 2400–0800. Consultants are not permitted to have other clinical responsibilities during this time. Current call volumes are around 14 per day and are still increasing. The service has been associated with safer and faster thrombolytic treatment, and lowered mortality [30]. Since 2021, the VST has expanded to cover three Tasmanian hospitals.

### The New Zealand National Hyper-Acute Stroke Program

New Zealand is a country the size of the United Kingdom but with a population of only 5.2 million people, of whom 2.3 million live in the three main cities (Auckland, Wellington and Christchurch). Population density elsewhere is very low, with many small and dispersed rural communities and other urban centres comprising populations under 200,000. The health system has a publicly funded hospital system providing all hyper-acute specialist stroke care, with primary care and some specialist and rehabilitation services provided privately.

In response to challenges identified in a 2009 nationwide stroke audit [31], the New Zealand (NZ) Ministry of Health funded the establishment of a clinician-led National Stroke Network, operated initially by the New Zealand Stroke Foundation. This group set stroke KPIs, developed nationally consistent stroke treatment protocols, and defined minimum stroke service standards.

In 2015 centralised stroke reperfusion data reporting was mandated [32] and a low-cost REDCap based national stroke register was implemented [33]. In 2016 the Central Region piloted the first NZ Telestroke network [34] with subsequent spread to other regions across the country, resulting in 90% of New Zealand stroke hospitals now being supported by remote experts when making thrombolysis treatment decisions employing four hubs and 21 spokes.

All these measures were associated with consistent improvements in reperfusion rates and treatment times [35]. In response to the publication of the pivotal EVT trials in 2015 and 2016, an EVT sub-group was formed. Based on population density and current reperfusion volumes, this group developed a national EVT strategy recommending that only three centres would be established to optimise volumes per centre, promote sustainable 24/7 neurointerventionalist rosters, and optimise patient transport times (Figure S2) with a view to add further centres once the initial three were fully operational 24/7. Around the same time the Greater Auckland (NZ’s largest urban region with 1.4 million people) implemented an EVT bypass model akin to the London HASU model although bypassing only potential EVT patients, using the PASTA score [35] and with limited operating hours.

In response to the nationally-developed EVT strategy an entirely new work programme was begun – separate from the NSN which then shifted its focus more toward sub-acute stroke care, rehabilitation, and prevention. The new programme started off with sector-wide consultation, including consumers, to confirm the terminology for EVT (which then in NZ changed to ‘Stroke Clot Retrieval (SCR)’ largely driven by consumer voice), confirm centre location, service specifications, and implementation priorities [36]. This resulted in the establishment of a multidisciplinary steering group, five sub-groups (imaging, neurointervention, telestroke, pre-hospital, and inter-hospital transport), and a consumer panel (with nearly 50% indigenous and 30% rurally based consumers) to implement the plan. The outputs included nationally agreed pre-hospital and inter-hospital transport policies, SCR referral criteria, telestroke service standards, expansion and refinement of KPIs – including patient-focussed outcomes, agreed imaging requirements, a national neurointervention fellowship, neurointervention training requirements, and consumer resources to aid in consenting and provide additional patient and family support [36]. The programme finished in early 2023 with the recommendation that a single national Hyper-Acute Stroke Service ought to be established to eradicate any remaining regional variation and inequities, enhance service sustainability, drive excellence, and reduce inefficiencies. This incorporated application for a single CT perfusion imaging software for all NZ stroke hospitals. A business case in support of this is currently being evaluated by the government. Measures implemented to date have resulted in a reduction in service variability, access inequities, and a 2023 national reperfusion rate of 18.4% (personal communication from Alicia Tyson to Anna Ranta 2 -7–2024 via email) with reduction in variation already noted in 2022 [37]. However, the systems remain vulnerable and without additional funding and progression to a national service there is significant concern around long-term sustainability.

Additional opportunities currently explored in NZ include countrywide roll-out of telestroke-facilitated ambulance triage, which demonstrated superiority of telestroke assessment for reperfusion eligibility versus ambulance stroke recognition scales, [38] national procurement of a single workflow tool, and support to the Pacific Islands [37].

### The South Australia/Northern Territory Telestroke Service – a geographically dispersed integrated hub and spoke service.

South Australia and the Northern Territory are sparsely populated, with a combined 2 M people dispersed over 2.3Mkm^2^, an area larger than Spain, Portugal, France, Italy, Germany and Poland combined (similar to a quarter of the United States of America). Approximately 75% of these 2 M people live in the greater Adelaide area [39] (11,000km^2^), and a further 140,000 in the greater Darwin area, 2624 km to the north. No neurologists (let alone vascular neurologists) reside outside of Darwin and Adelaide.

Adelaide has undergone centralisation of stroke services similarly to the London model, progressively from 2010. At that stage there were three hospitals with 24/7 stroke units, the Royal Adelaide (RAH) (the Comprehensive Stroke Centre), the Queen Elizabeth (QEH) (a Primary Stroke Centre with EVT capability) and Flinders Medical Centre (FMC – also a Primary Stroke Centre with EVT capability). The QEH was around 10 min’ drive from the RAH, and FMC 20 min (Figure S3). In 2011, a stroke thrombolytic service was established (0800–1600 Monday to Friday) at the Lyell McEwin Hospital (LMH), a hospital 30 min north of the RAH in a previously unserved area of rapid population growth. In 2013 an LMH Stroke Unit was established, and in 2016 the QEH and RAH stroke units merged. In 2016 a bespoke stroke data platform was introduced for all metropolitan admissions, feeding into the AuSCR. In 2017, as the RAH was the only site with all-hours on-site CT, EVT and overnight (2000–0800) ‘code stroke’ was centralised to the RAH, while ‘code stroke’ was expanded to 0800–2000 daily at the LMH. In 2022, using modelling based on door-to-needle and door-in door-out (DIDO) times at FMC and the LMH [40], patients with highly probable LVO symptoms in the ambulance (as assessed by the ‘Arm Chat Tap’ test) [41] bypassed directly to the RAH.

From June 2018 a Telestroke service to South Australian Regional sites was introduced. Using Cisco Jabber software, and cameras wall-mounted in Emergency departments, acute stroke (with symptoms less than 24 h duration) support has been provided 24/7 to all 61 small regional hospitals, including 4 hospitals with multimodal imaging and a further 4 with CT and CT angiogram capability. Patients are assessed for tenecteplase thrombolytic eligibility (administered under visual supervision to ensure correct dosing), and transferred if eligible for thrombectomy, vascular surgery, neurosurgery and stroke unit care. Logistic transfer advice is provided for non-neuroimaging sites. All stroke patients are offered care in a Stroke Centre or one of three Stroke Capable Regional General Hospitals. Stroke Unit Certification is being rolled out to both metropolitan and regional sites. An integrated Telemedicine platform ‘Zeus’ has been implemented, with data automatically collated for quality improvement. Both the service and the subsequent enhancement enabled by the Zeus platform have been associated with improved metrics and outcomes [42, 43].

Recently the Telestroke service has provided interstate services to Alice Springs Hospital (1313 km distant) and facilitated expansion of a thrombolytic service to out-of-hours in Darwin. Selected cases from Alice Springs and Darwin are retrieved for thrombectomy from both locations, given the expansion of the thrombectomy window to 24 h, especially in ‘slow progressors’ [44].

RAH acute stroke and Telestroke rosters are largely combined. Statewide reperfusion rates are nation-leading [45]. Increasing call volume, especially overnight, has led to the introduction of ‘stroke nights’ rostering of the 8 vascular neurology consultants for a week at a time, without scheduled clinical duties the following day.

## Common and disparate elements of well-functioning stroke systems

### A clinician-led, consensus-based approach to system improvement

All the system examples share the common theme of being led and developed by expert clinicians who were able to build consensus. While the London HASU change model was the most government-initiated, the design and implementation was clinician-led through a group of managed clinical networks. System-wide improvement in the other three examples were clinician-led but matched by government support. Population-wide service coverage and optimisation is substantially more difficult to achieve in countries where hospital and pre-hospital services are supplied by a multiplicity of providers, such as in the United States of America (USA).

The marked disparity in stroke treatment rates between countries is not readily explained by differing healthcare system designs [11]. While voluntary, less centralised planning approaches such as the American Heart Association Get With the Guidelines (GWTG) program can also be associated with improved reperfusion rates and metrics [46] in universal health care systems, combining top-down authority with bottom-up clinical leadership is critical for success [47].

### Rational system design based on patient demand and healthcare supply

Most famine in the modern era does not primarily result from global food shortage, but from inequitable and inefficient distribution [48]. So, too, unequal access to timely stroke care in a given country is not only due to a shortage of stroke resources (both hospitals and clinicians) but can be significantly mitigated by improved organisation. This is true both of thrombolytic services and EVT [49].

The four stroke systems outlined all have redistributed consultant-led services, in some instances creating new services in areas of need, and in others closing services where such services were either unjustified (as being located too close to another identical service) or inefficient. Further, these systems have used both technology and improved patient transport logistics to bring the doctor to the patient (through telemedicine to another hospital or into the ambulance) and when needed the patient to the doctor. Even with extremely long transfer distances, the net monetary benefit from successful reperfusion in the setting of large vessel occlusion [50] makes such long-distance transfers worthwhile [24].

Current ongoing system design issues include the best utilisation of MSUs and the place of ambulance bypass protocols for patients with probable LVO. While MSUs may be justifiable in large, dense population centres, their utility in smaller, more dispersed cities is uncertain and requires more cost-effectiveness research. Bypass of non-EVT centres to thrombectomy centres was not associated with improved outcomes in the RACECAT trial [51], however workflow metrics were extremely efficient in the Catalonia region, and results may differ in regions with less efficient door-needle and DIDO times at PSCs.

### Centralisation of services

All service examples employed centralisation to improve patient care. The London HASU model was based on pre-existing data suggesting the centralisation benefits, from other condition such as major trauma [52], but greatly strengthened the evidence base for such centralisation in stroke [25, 26], providing a template for service reorganization in Adelaide, Auckland, and elsewhere [53].

There is a wide and increasing body of evidence linking volume with treatment quality and stroke outcome. For example, studies of both EVT in the USA [54] and thrombolytic therapy in the UK [55] have shown that treatment times are fastest where annual volumes exceed 100 cases. However, challenges of centralisation include potential deskilling and demoralisation of staff, community and clinician resistance to closing services, lessening of training opportunities, travel barriers for relatives of patients treated far from home, and difficult or delayed repatriation back to ‘home base’ hospitals [47] and ongoing work is required to address these challenges.

### The facilitating role of data-based quality improvement and stroke system frameworks.

All systems discussed are buttressed by a foundation of stroke unit care, one requirement of which is use of data for quality improvement [56].National and international guidelines strongly support a network-based approach to stroke system development, in which there are strong linkages between Telestroke Centres, Primary Stroke Centres and Comprehensive Stroke Centres [57–59].

This routine use of data for quality improvement has also facilitated numerous research publications (see above), both evaluating and demonstrating the benefit of system developments. An international exemplar has been the GWTG program, which has been able to identify and rank the clinical elements which contribute most to improved reperfusion metrics [60].

The rational and routine use of data for quality improvement facilitates a ‘Learning Healthcare System’[61, 62], within which interventions can be iteratively developed or altered. As long as the data elements themselves are also flexible, this model can both adapt to and shape improvements in stroke care, while evaluating and maximising cost-effectiveness.

An important contributor to a Learning Healthcare System is the identification of key priorities, and the setting of targets. The potential areas for quality improvement are many, however rank-ordering of priorities guided by probable health economic impact, can prioritise KPIs. Again, an example of successful target-setting is the GWTG Target stroke initiative, which has focused on the clear patient benefits of improving reperfusion timelines [63, 64]. GWTG has been highly successful in setting reperfusion goals in participating US hospitals [65]. Subsequently, target setting has been adopted internationally, for instance in the Stroke Action Plan for Europe [66], and the Australian 30/60/90 National Stroke Targets [67].

### Future opportunities

The emerging evidence of ambulance-based blood pressure lowering in intracerebral haemorrhage [23] will increase the impetus for mobile stroke units in urban areas. This will be facilitated by the development of novel, lightweight imaging devices [68], which may reduce the need for onboard stroke neurologists and radiographers, who will increasingly be able to provide telestroke services to mobile stroke units and possibly stroke air ambulances. This telestroke MSU model will be supported by evidence of tenecteplase superiority over alteplase, which is logistically easier to administer under remote supervision due to bolus administration [69]. Ambulance based telestroke could be integrated with a regional telestroke service for efficiency, as is planned in New Zealand.

Telestroke triage could not only be brought to normal ambulances to help triage patients and speed reperfusion in metropolitan areas [27], it could also be utilized by emergency telephone call takers. Facilitated by the wide dissemination of smartphones, secure smartphone video calls can now be established with primary responders, for instance through the cardiac arrest GoodSAM Instant-on-scene™ platform [70]. The widespread implementation of hyper-acute stroke workflow tools such as Zeus[43] or similar tools is also likely going to increasingly optimise efficiency.

Robotic endovascular neurointervention may allow remote thrombectomy in regionally-located patients by centrally-located neurointervention teams [71]. Closing the expertise gap in regional areas is not just important for hyperacute care, but also in the provision of post-acute and stroke rehabilitation services. The VST is currently developing remote stroke neurologist post-acute support through the ‘Bridging the Urban and regIonaL Divide in Stroke care’ (BUILDS) program [72]. Technological advances may further strengthen the evidence base for telerehabilitation [73], not only facilitating earlier discharge home, but also enhancing provision of rehabilitation to regional and remote areas.

## Conclusions

Rapid advances in stroke care have required evolution of time-critical hyperacute stroke services, and innovative workflow responses. These have been most successful where top-down government and bottom-up clinician initiatives have aligned, permitting the rationalisation and redesign of stroke care systems, assisted by advances in neuroimaging and telecommunications. Where possible, services should be centralised to maximise stroke expertise, guided by modelling of distances and transport times. A mix of centralisation, and telemedicine support of more remote sites, guided by insights from universally collected stroke data and focused by stroke KPI targets, will maximise beneficial patient outcomes.

## **Key References**

- Rudd AG, Bladin C, Carli P, De Silva DA, Field TS, Jauch EC, et al. Utstein recommendation for emergency stroke care. Int J Stroke. 2020;15(5):555-64. **Global initiative to streamline and standardise pre-hospital care.**
- Sarraj A, Kleinig TJ, Hassan AE, Portela PC, Ortega-Gutierrez S, Abraham MG, et al. Association of Endovascular Thrombectomy vs Medical Management With Functional and Safety Outcomes in Patients Treated Beyond 24 Hours of Last Known Well: The SELECT Late Study. JAMA Neurol. 2023;80(2):172-82. **Important paper suggesting benefit of EVT in selected patients beyond 24 hours.**
- Xiong Y, Campbell BCV, Schwamm LH, Meng X, Jin A, Parsons MW, et al. Tenecteplase for Ischemic Stroke at 4.5 to 24 Hours without Thrombectomy. New England Journal of Medicine. 2024. **Key recent paper suggesting substantial benefit of extended window thrombolysis in centres without ready access to thrombectomy. Important for lower income countries with poor EVT access, and for ‘bolus and ship’ primary stroke centres, especially if long transport times are expected.**
- Ma L, Hu X, Song L, Chen X, Ouyang M, Billot L, et al. The third Intensive Care Bundle with Blood Pressure Reduction in Acute Cerebral Haemorrhage Trial (INTERACT3): an international, stepped wedge cluster randomised controlled trial. The Lancet. 2023;402(10395):27-40. **Bundling acute ICH interventions together.**
- Pradilla G, Ratcliff JJ, Hall AJ, Saville BR, Allen JW, Paulon G, et al. Trial of Early Minimally Invasive Removal of Intracerebral Hemorrhage. New England Journal of Medicine. 2024;390(14):1277-89. **The first RCT clearly demonstrating the benefit of ICH evacuation. If replicated, substantial system of care reorganisation will be required.**
- Li G, Lin Y, Yang J, Anderson CS, Chen C, Liu F, et al. Intensive Ambulance-Delivered Blood-Pressure Reduction in Hyperacute Stroke. New England Journal of Medicine. 2024. **Key RCT further strengthening the evidence for ultra-early blood pressure lowering in ICH, within 2 hours of onset, in the ambulance. The trial overall was neutral, seemingly with significant benefit in ICH balancing significant harm in acute ischaemic stroke.**
- Garcia-Esperon C, Wu TY, Carraro do Nascimento V, Yan B, Kurunawai C, Kleinig T, et al. Ultra-Long Transfers for Endovascular Thrombectomy-Mission Impossible?: The Australia-New Zealand Experience. Stroke. 2023;54(1):151-8. **This paper demostrates that very long-distances transfer (up to 2600km) for thrombectomy is feasible. In advanced wealthy country, distance should be no barrier to EVT for LVO stroke for selected patients. Given EVT cost-benefits, systems of care need to evolve to make this logistically feasible.**
- Morris S, Hunter RM, Ramsay AI, Boaden R, McKevitt C, Perry C, et al. Impact of centralising acute stroke services in English metropolitan areas on mortality and length of hospital stay: difference-in-differences analysis. BMJ. 2014;349:g4757. **Not a recent paper, but a key paper demonstrating the benefits of stroke service centralisation, benefit not seen when care of only selected patients was centralised.**
- Holodinsky JK, Williamson TS, Demchuk AM, Zhao H, Zhu L, Francis MJ, et al. Modeling Stroke Patient Transport for All Patients With Suspected Large-Vessel Occlusion. JAMA Neurol. 2018;75(12):1477-86. **This landmark modelling paper has served as the foundation for many stroke system reorganisation plans.**
- Albers GW. Late Window Paradox. Stroke. 2018;49(3):768-71. **Key concept paper, explaining elegantly how time is brain for all, but especially so for some.**
- Stroke Foundation. National Stroke Audit – Acute Services Report 2021. Melbourne, Australia2021.ss Man S, Solomon N, Mac Grory B, Alhanti B, Saver JL, Smith EE, et al. Trends in Stroke Thrombolysis Care Metrics and Outcomes by Race and Ethnicity, 2003-2021. JAMA Network Open. 2024;7(2). **This paper demostrates the sustained, continuous improvement in door-thrombolytic times in the United States of America, fostered by the GWTG program.**
- Kunz WG, Almekhlafi MA, Menon BK, Saver JL, Hunink MG, Dippel DWJ, et al. Public Health and Cost Benefits of Successful Reperfusion After Thrombectomy for Stroke. Stroke. 2020;51(3):899-907. **Rather than calculating the ‘cost benefits’ of intervention, this paper applies a quality-adjusted life year ‘willingness to pay’ approach to the HERMES EVT data, to demonstrate the substantial net monetary benefit of successful reperfusion. This paper serves as a foundation to persuade health organisations to pay for fast and effective reperfusion initiatives.**
- Perez de la Ossa N, Abilleira S, Jovin TG, Garcia-Tornel A, Jimenez X, Urra X, et al. Effect of Direct Transportation to Thrombectomy-Capable Center vs Local Stroke Center on Neurological Outcomes in Patients With Suspected Large-Vessel Occlusion Stroke in Nonurban Areas: The RACECAT Randomized Clinical Trial. JAMA. 2022;327(18):1782-94. **This somewhat surprisingly negative trial raises caveats for large vessel occlusion bypass, especially when transfer distances are long, and prinary stroke centre door-thrombolytic times are short. INTERACT-3 and -4 provide a further explanation of the neutral trial - potential harm from delayed blood pressure lowering in acute ICH.**
- Bryndová L, Bar M, Herzig R, Mikulík R, Neumann J, Šaňák D, et al. Concentrating stroke care provision in the Czech Republic: The establishment of Stroke Centres in 2011 has led to improved outcomes. Health Policy. 2021;125(4):520-5. **Although not covered here due to space concerns, the Czech Republic provides an exemplar case study in nation-wide stroke quality improvement.**
- Nogueira RG, Haussen DC, Smith EE, Sun JL, Xian Y, Alhanti B, et al. Higher Procedural Volumes Are Associated with Faster Treatment Times, Better Functional Outcomes, and Lower Mortality in Patients Undergoing Endovascular Treatment for Acute Ischemic Stroke. Ann Neurol. 2023. **In this very large GWTG cohort higher volume EVT sites had faster door-thrombolytic and door-first pass times, and higher reperfusion rates, which translated to lower mortality and better functional outcome at discharge.**
- Dusenbury W, Mathiesen C, Whaley M, Adeoye O, Leslie-Mazwi T, Williams S, et al. Ideal Foundational Requirements for Stroke Program Development and Growth: A Scientific Statement From the American Heart Association. Stroke. 2023;54(4). **Excellent recent summary of the key components of a high-quality stroke system of care.**
- Cadilhac DA, Bravata DM, Bettger JP, Mikulik R, Norrving B, Uvere EO, et al. Stroke Learning Health Systems: A Topical Narrative Review With Case Examples. Stroke. 2023;54(4):1148-59. **Comprehensive narrative overview of applying the Learning Health System concept to Stroke.**
- Cadilhac DA, Bravata DM, Bettger JP, Mikulik R, Norrving B, Uvere EO, et al. Stroke Learning Health Systems: A Topical Narrative Review With Case Examples. Stroke. 2023;54(4):1148-59. **Comprehensive narrative overview of applying the Learning Health System concept to Stroke.**
- Xian Y, Xu H, Smith EE, Saver JL, Reeves MJ, Bhatt DL, et al. Achieving More Rapid Door-to-Needle Times and Improved Outcomes in Acute Ischemic Stroke in a Nationwide Quality Improvement Intervention. Stroke. 2022;53(4):1328-38. **Key door-thrombolytic quality improvement paper.**
- Parsons MW, Yogendrakumar V, Churilov L, Garcia-Esperon C, Campbell BCV, Russell ML, et al. Tenecteplase versus alteplase for thrombolysis in patients selected by use of perfusion imaging within 4·5 h of onset of ischaemic stroke (TASTE): a multicentre, randomised, controlled, phase 3 non-inferiority trial. The Lancet Neurology. 2024. **This phase 3 tenecteplase trial with the meta-analysis performed in the paper demostrate a number needed to treat of 25 for superior excellent outcome with tenecteplase, facilitating the system-wide shift to this agent rather than alteplase.**
- ter Avest E, Lambert E, de Coverly R, Tucker H, Griggs J, Wilson MH, et al. Live video footage from scene to aid helicopter emergency medical service dispatch: a feasibility study. Scand J Trauma Resusc Emerg Med. 2019;27(1). **Adding ‘smartphone’ primary responder video to initial call-taker interactions may prove beneficial in shorting dispatch times, and ensuring mbolie stroke units attend high probability stroke cases.**
- Mendes Pereira V, Cancelliere NM, Nicholson P, Radovanovic I, Drake KE, Sungur J-M, et al. First-in-human, robotic-assisted neuroendovascular intervention. Journal of NeuroInterventional Surgery. 2020;12(4):338-40. **In larger regional centres without neurointerventionalists, but with angiography suites, remote robotic-assisted thrombectomy may prove effective and cost-effective.**
- Mendes Pereira V, Cancelliere NM, Nicholson P, Radovanovic I, Drake KE, Sungur J-M, et al. First-in-human, robotic-assisted neuroendovascular intervention. Journal of NeuroInterventional Surgery. 2020;12(4):338-40. **In larger regional centres without neurointerventionalists, but with angiography suites, remote robotic-assisted thrombectomy may prove effective and cost-effective.**

## Supplementary Information

Below is the link to the electronic supplementary material.

Supplementary Material 1.

## Data Availability

No datasets were generated or analysed during the current study.
