# Supplementary material for: Hyper-Acute Stroke Systems of Care and Workflow
Source: Curr Neurol Neurosci Rep. 2024 Aug 16;24(10):495–505. doi: 10.1007/s11910-024-01367-6 (PMC11415429; doi:10.1007/s11910-024-01367-6)
Supplement: Supplementary file 1 — Supplementary Material 1. [file 11910_2024_1367_MOESM1_ESM.docx]

Figure S1 The Victorian Stroke Telemedicine Service


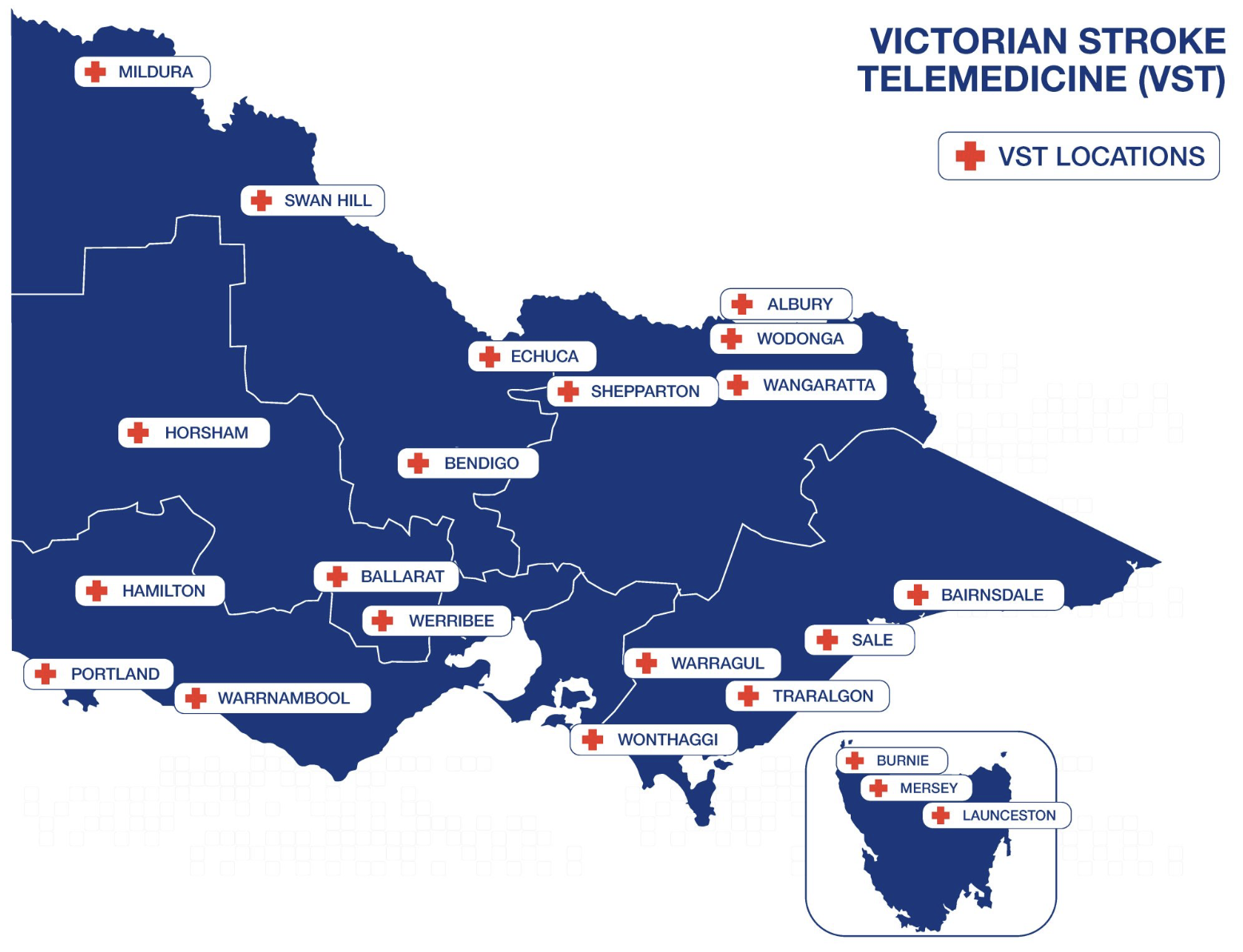


The VST provides an acute telestroke service to 19 Victorian sites and three sites in Tasmania.[1]

Figure S2 Endovascular thrombectomy catchments for the 3 Comprehensive Stroke Centres in New Zealand


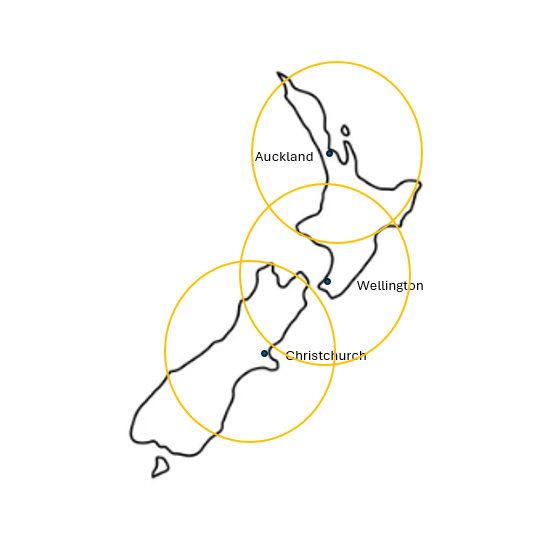


EVT centres in Auckland, Wellington, and Christchurch. Circles indicate helicopter transport times of <2 hours.

Figure S3 Metropolitan-wide Stroke System Reorganisation in Adelaide, Australia


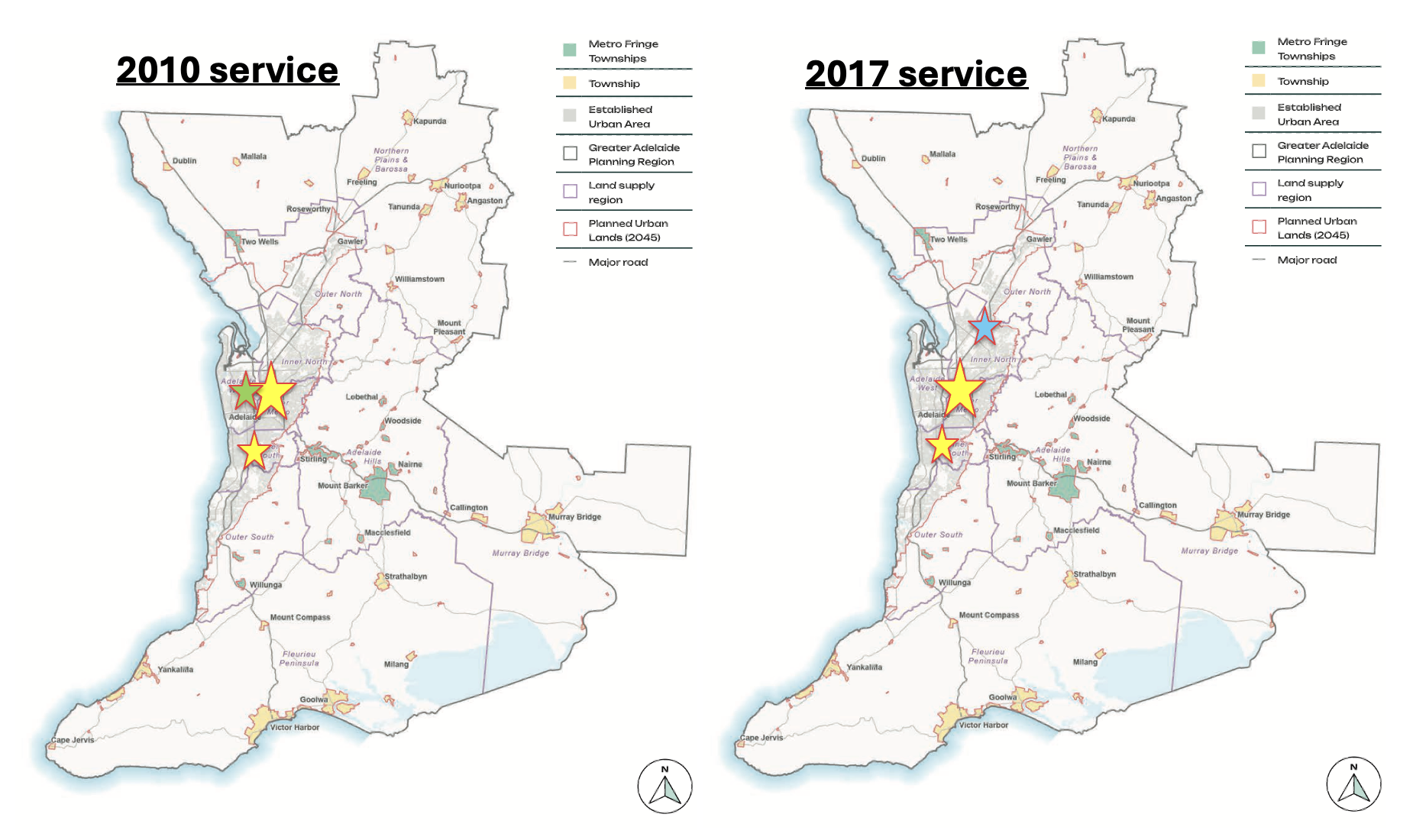


Map of the greater Adelaide area (population 1.5 Million),[2] with stroke centres superimposed, comprising the Adelaide metropolitan area (central) and surrounding smaller towns, up to 100km distant. No town outside of Adelaide metropolitan area has a population of more than 20,000 people. All patients with stroke symptoms detected by ambulance in this region are brought directly to metropolitan stroke units. In 2010 there were three stroke units, the Royal Adelaide Hospital (RAH) (large star), the Queen Elizabeth Hospital (QEH) (green star) and Flinders Medical Centre (FMC) (small star). By 2017, the RAH and QEH had merged, a Primary stroke centre had opened at the Lyell McEwin Hospital (LMH) (blue star) and endovascular neurointervention was centralised to the RAH. Thrombolysis was provided 0800-2000 at FMC and the LMH, but 2000-0800 all overnight ‘code stroke’ ambulance transfers drained to the RAH.

Supplementary references:

1. Ambulance Victoria: Victorian Stroke Telemedicine. <https://www.ambulance.vic.gov.au/about-us/our-services/victorian-stroke-telemedicine/> (2024). Accessed July 17 2024.

2. Government of South Australia: Greater Adelaide regional plan: discussion paper summary. <https://plan.sa.gov.au/__data/assets/pdf_file/0005/1259627/Greater-Adelaide-Regional-Plan-Discussion-Paper-Summary.pdf> (2023). Accessed July 17 2024.
